# Disclosing Critical Voice Features for Discriminating between Depression and Insomnia—A Preliminary Study for Developing a Quantitative Method

**DOI:** 10.3390/healthcare10050935

**Published:** 2022-05-18

**Authors:** Ray F. Lin, Ting-Kai Leung, Yung-Ping Liu, Kai-Rong Hu

**Affiliations:** 1 Department of Industrial Engineering and Management, Yuan Ze University, Taoyuan 32003, Taiwan; karenhu0205@gmail.com; 2 Department of Radiology, Taoyuan General Hospital, Ministry of Health and Welfare, No. 1492, Zhongshan Rd., Taoyuan City 33004, Taiwan; hk8648@tmu.edu.tw; 3 Graduate Institute of Biomedical Materials and Tissue Engineering, College of Biomedical Engineering, Taipei Medical University, Taipei 11031, Taiwan; 4 Department of Industrial Engineering and Management, Chaoyang University of Technology, Taichung 413310, Taiwan; ypliu@cyut.edu.tw

**Keywords:** depression, insomnia, openSMILE, voice feature, computer-aided diagnosis

## Abstract

**Background:** Depression and insomnia are highly related—insomnia is a common symptom among depression patients, and insomnia can result in depression. Although depression patients and insomnia patients should be treated with different approaches, the lack of practical biological markers makes it difficult to discriminate between depression and insomnia effectively. **Purpose:** This study aimed to disclose critical vocal features for discriminating between depression and insomnia. **Methods:** Four groups of patients, comprising six severe-depression patients, four moderate-depression patients, ten insomnia patients, and four patients with chronic pain disorder (CPD) participated in this preliminary study, which aimed to record their speaking voices. An open-source software, openSMILE, was applied to extract 384 voice features. Analysis of variance was used to analyze the effects of the four patient statuses on these voice features. **Results:** statistical analyses showed significant relationships between patient status and voice features. Patients with severe depression, moderate depression, insomnia, and CPD reacted differently to certain voice features. Critical voice features were reported based on these statistical relationships. **Conclusions:** This preliminary study shows the potential in developing discriminating models of depression and insomnia using voice features. Future studies should recruit an adequate number of patients to confirm these voice features and increase the number of data for developing a quantitative method.

## 1. Introduction

### 1.1. Depression vs. Insomnia

Depression and insomnia are both prevalent disorders that are highly related. Lim, et al. [1] evaluated the prevalence of depression in different countries between 1994 and 2014 and found that the aggregated point, one-year, and lifetime prevalence of depression were 12.9, 7.2 and 10.8%, respectively. On the other hand, many studies reported that chronic insomnia affects, approximately, from 5 to 30% of the population [2,3,4,5,6,7]. Research has shown a strong relationship between insomnia and depression [8,9,10,11,12]. However, the exact nature of the relationship is complex [9]. Insomnia, defined as sleep difficulty, can occur independently or due to other problems, including psychological factors (e.g., stress), physical factors (e.g., chronic pain), and other possibilities. Many studies considered insomnia a symptom of depression, but recent studies have found insomnia is also a risk factor for the development of depression [12,13,14]. Depressed patients with insomnia as a symptom could be treated with antidepressant medication or cognitive therapy [15]. However, insomnia patients should be treated differently from depression patients. Recent studies show that cognitive behavioral therapy for insomnia is highly effective compared to other psychological interventions [16,17]. Nonetheless, applying depression treatments to insomnia patients may cause a relapse of depression [13]. Hence, it is critical to have accurate diagnoses so that these two types of patients can be treated well.

### 1.2. Diagnoses of Depression and Insomnia

It has been pointed out that both diagnoses of depression and insomnia are difficult tasks. To diagnose and assess depression, one should rely on subjective behavior measures, such as self-reports or family reports and clinical interviews [18,19]. Commonly used assessment tools are the Hamilton Rating Scale for Depression [20] and the Beck Depression Index [21]. An expert practitioner subjectively performs these rating scales based on a patient’s mental state, driven by interviews and self-report experiences [22]. However, the assessment is costly, time-consuming, and often requires patients’ presence at the clinic.

On the other hand, similar difficulties were reported while diagnosing insomnia. Schramm, et al. [23] pointed out practical issues in structured clinical interviews, which are ineffective and time-consuming. Although tools (e.g., the Insomnia Severity Index, Athens Insomnia Scale, and Pittsburg Sleep Quality Index) have been developed for screening insomnia, evidence shows that insomnia is still under-recognized, underdiagnosed, and undertreated [24,25]. Consequently, it is necessary to develop a more systematic way of measuring and quantifying depression and insomnia within and across clinical sessions for a more effective and efficient diagnosis.

### 1.3. Speech Voice May Help Diagnoses

Human speech voice correlates with the emotional state, so using voice may aid in developing a quantitative method for distinguishing depression patients from insomnia patients [19]. It is well-known that gender influences speech differences. For example, compared to males, females have a relatively higher pitch [26,27], greater voice quality [27], and a larger pitch range [28]. Except for the changes due to gender, slight physiological and cognitive changes can also produce acoustic changes [29,30,31]. Research on emotional speech has shown that emotion affects prosodic and spectral speech characteristics [32]. The speech production and acoustic quality of the speech are affected by depression caused by cognitive and physiological changes [31].

Although studies have shown that depression leads to speech differences and using speech to distinguish depression is feasible [31,32,33,34,35,36], there is little evidence showing that insomnia causes differences in voice. In contrast, Heydarifard and Krasikova [37] found no association between insomnia and next-day prohibitive voice. While depression caused by cognitive and physiological changes affects speech and insomnia has no effects on speech, it is reasonable to hypothesize that voice features exist to discriminate between depression and insomnia. However, to the authors’ best knowledge, few studies have directly reported speech differences between depression and insomnia.

### 1.4. Research Objective

While diagnosing depression and insomnia is difficult, and research gaps exist in determining their effects on voice, this preliminary study aimed to test the effects of depression and insomnia on voice features. With a limited number of participants, we expected that certain voice features would show the differences between depression and insomnia. If so, this preliminary study could confirm the possibility of executing the following research with an adequate number of patients for data collection and, hence, develop a quantitative method to discriminate between depression and insomnia.

## 2. Materials and Methods

### 2.1. Participants and Clinical Assessment

Twenty-four patients who visited the Department of Radiology, the Taoyuan General Hospital, Ministry of Health and Welfare, from July 2018 to September 2018, volunteered to participate in this study. They belonged to different outpatient departments and suffered from psychological diseases, sleep difficulty, or chronic pain disorders. Outpatient physicians recruited these participants by inviting every visiting patient who presented with the above symptoms during off-peak clinic hours. To categorize these participants, they were required to fill out the Hamilton Depression Rating Scale [38] questionnaire and other clinical assessments by our clinical research physician. As suggested by Zimmerman, et al. [39], score thresholds of 17 and 23 were first used to determine groups of moderate depression (17–23) and severe depression (23–65).The rest of the patients whose scores were less than 17 were defined as a non-depression group. The clinical research physician further categorized them according to their clinical features. As a result, there were six severe-depression patients (two females/four males), four moderate-depression patients (two females/two males), ten insomnia patients (six females/four males), and four patients (one females/three males) with chronic pain disorders (CPD; e.g., myalgia and knee pain).

### 2.2. Collection and Preprocessing of the Speech Voice

After the informed consent, the participants moved to a quiet room located in the hospital to record their speech voice data. The surrounding noise in the rooms was maintained under 45 dB. The participants followed the experimenter’s instructions to read out the 21 questions of the Chinese version of the Beck Anxiety Inventory [40]. A microphone connected to a personal computer collected voice data while the participant was reading these questions.

The speech data were saved as a single channel, MP3 files, sampled at 44.1 kHz, 8-bit.The recorded MP3 files underwent several preprocesses using a voice processing software, Audacity^TM^. First, the MP3 files were converted to WAV files. Second, irrelevant proportions of sound clips (e.g., cough, sneeze, chair moving, etc.) were cut out. Third, background noises were eliminated. Last, the files were cut into clips as the participants read a single question of the CBAI.

### 2.3. Voice Features Extraction and Statistical Analysis

After data preprocessing, an open-source software, openSMILE, IS09_eotion [41], was applied to extract 384 voice features. These features were calculated based on 16 descriptors, comprising root mean square (RMS), zero-crossing rate (ZCR), pitch frequency (F0), harmonics-to-noise ratio (HNR), and Mel-frequency cepstral coefficients (MFCC) 1–12. These original 16 descriptors (*d*) were then used to capture de-differentiated (partial differential) descriptors (*d’*), representing non-personalized features [42]. Next, the 12 statistic properties of the mean, standard deviation (SD), kurtosis, skewness, maximum and minimum values, maximum and minimum positions, and range, as well as two linear regression coefficients (offset and slope) with their mean square error (MSE), were computed for these 32 descriptors. The effects of participant status (i.e., severe-depression, moderate-depression, insomnia, and CPD) on these 384 voice features (16 × 2 × 12) were then assessed using analysis of variance (ANOVA). In addition to patient status, gender was also treated as an independent variable in analyses because gender is a critical factor that affects speech voice.

## 3. Results

ANOVA was performed on all the 384 voice features, using a model with status and gender as fixed effects and participant as a random effect nested within status and gender. As shown in Table 1, Table 2 and Table 3, there were many significant main effects and interaction effects of status and gender on voice features. While the focus is on patient status, all the main effects of status are depicted and detailed. For the main effects of gender and interaction effects of gender and status, the distributions of statistical effects were presented to provide the recommendations for selecting voice features due to the limited space.

### 3.1. Effects of Patient Status

**Figure 1:**
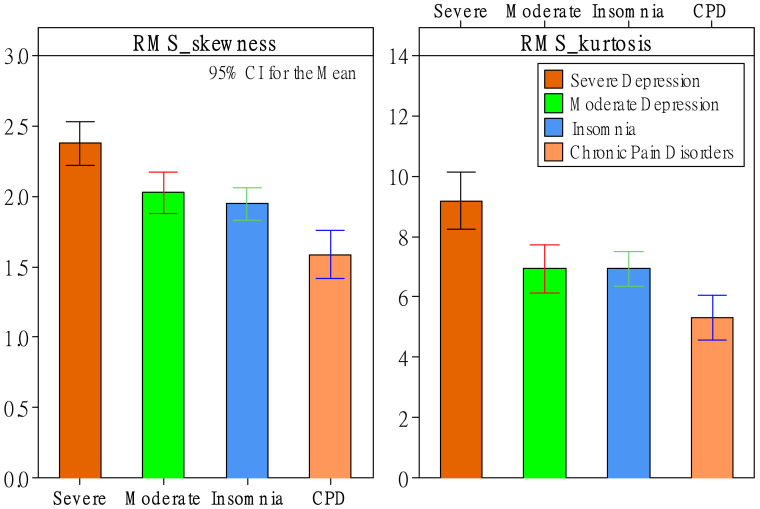
The effect of patient status on the root mean square (RMS).

**Figure 2:**
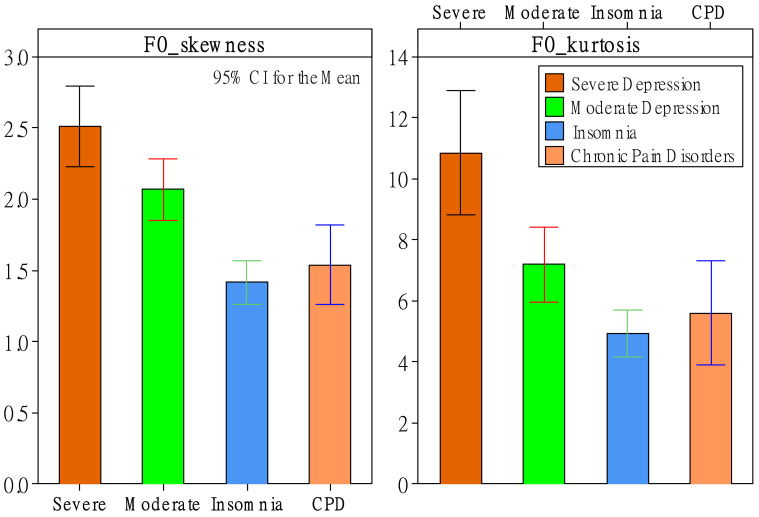
The effect of patient status on the pitch (F0).

**Figure 3:**
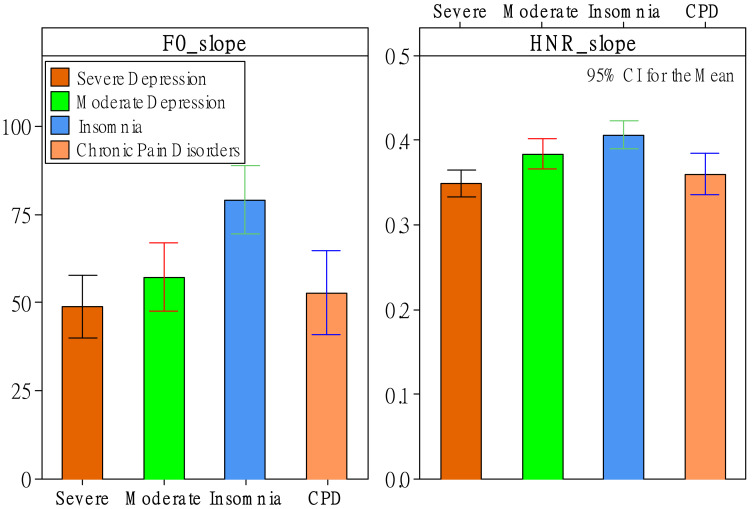
The effect of patient status on slopes of F0 and HNR.

Regarding RMS, ZCR, F0, and HNR, status had significant effects (*p*-value < 0.05) on six *d* features but zero *d’* features. Status had significant effects on skewness and kurtosis of original descriptors of RMS and F0. As shown in Figure 1, the severe-depression group had the greatest RMS-skewness and RMS-kurtosis values compared to the other three groups. This phenomenon was even more apparent for F0-skewness and F0-kurtosis values. As shown in Figure 2, depression patients had significantly greater F0-skewness and F0-kurtosis values than patients with insomnia and CPD. Moreover, the severe-depression group had significantly greater values than the moderate-depression group. Furthermore, status had significant effects on slopes of F0 and HNR. As shown in Figure 3, the insomnia group had the greatest slope values of F0 and HNR compared to the other three groups.

### 3.2. Effects of Patient Status on MFCC

**Figure 4:**
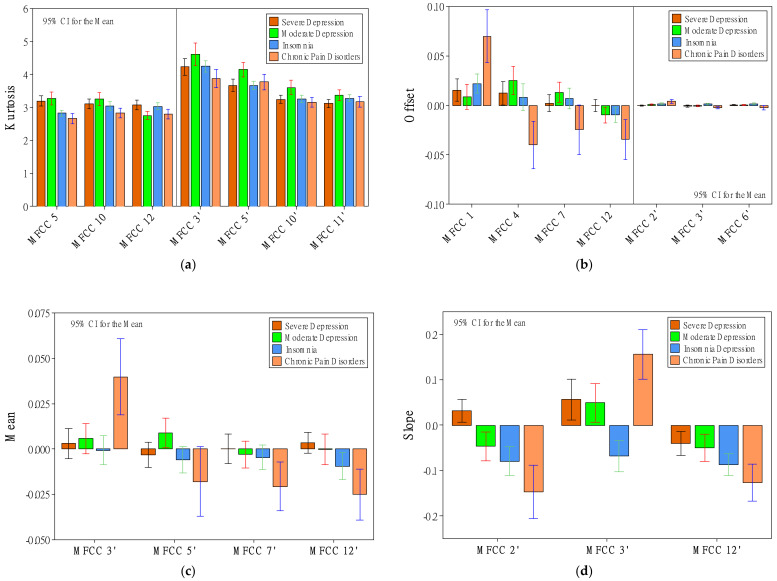

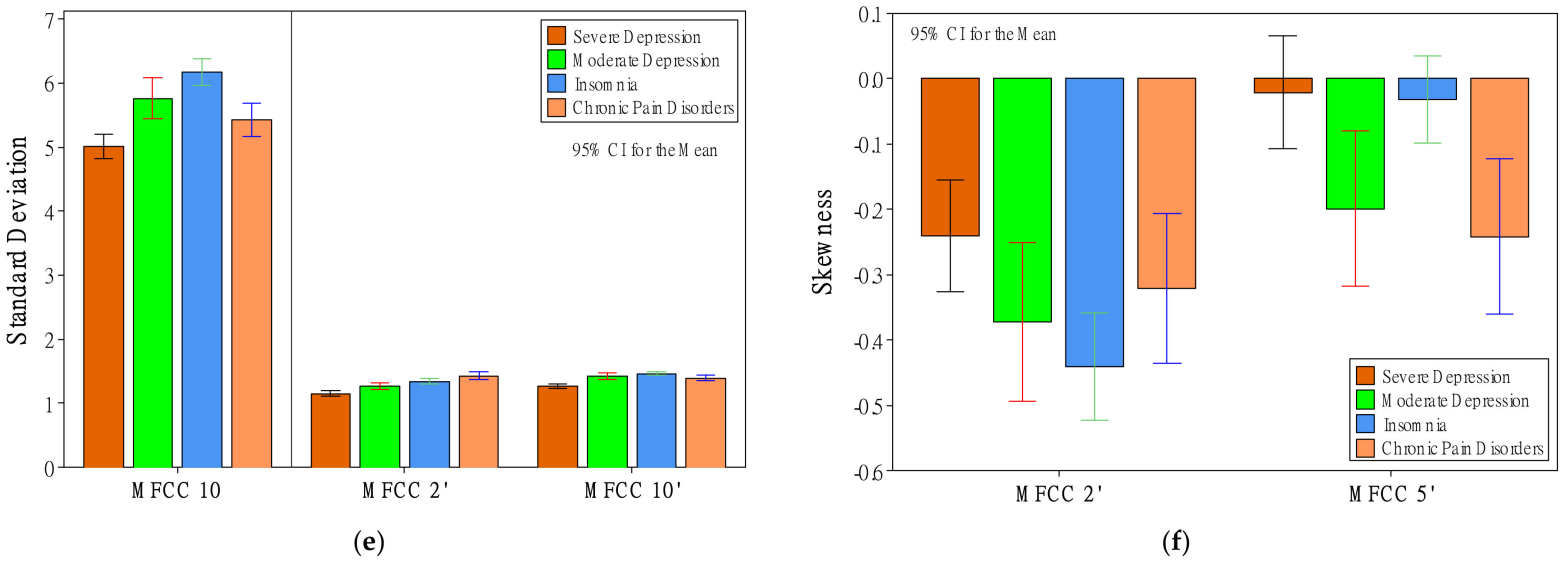
The effect of patient status on kurtosis (**a**), offset (**b**), mean (**c**), slope (**d**), SD (**e**), and skewness (**f**).

Regarding MFCC features, status had significant effects on 13 *d* features and 23 *d’* features. Kurtosis, again, was an essential feature in showing the differences among the patient groups. There were three significant status effects related to kurtosis found in *d* features (i.e., MFCCs 5, 10, and 12) and four in *d’* features (i.e., MFCCs 3′, 5′, 10′, and 11′). As shown in Figure 4a, as far *d* features are concerned, there was a trend in that depression patients had greater kurtosis values, especially MFCC 5, compared to groups of insomnia and CPD. Where *d’* features are concerned, the moderate-depression group had the greatest kurtosis values among the three groups. Another critical category of features was the offset of the regression coefficient. There were four significant status effects related to offset found in *d* features (i.e., MFCCs 1, 4, 7, and 12) and three in *d’* features (i.e., MFCCs 2′, 3′, and 6′). As shown in Figure 4b, the CPD group had significant differences from the other three groups. The group had the greatest offset values of MFCC 1 and MFCC 2′, and had the lowest offset values of MFCCs 4, 7, 12, 3′, and 6′. Worth noting is that mean and slope features showed the patient differences only in *d’* features, not *d* features. There were four significant relationships of mean features (MFCCs 3′, 5′, 7′, and 12), and three significant relationships of slope features (MFCCs 2′, 3′, and 12′). Regarding the mean features, as shown in Figure 4c, again, the CPD group had significant differences from the other three groups. It had the greatest mean value of MFCC 3′ and had the lowest mean values of MFCCs 5′, 7′, and 12′ in *d’* features. Regarding the slope features, as shown in Figure 4d, there was a trend in that depression patients had greater slope values of MFCCs 2′ and 12′ compared to the groups of insomnia and CPD. Again, the CPD group had significant differences from the other three groups. It had the greatest slope value of MFCC 3′ and the lowest slope values of MFCCs 2′ and 12′.

**Figure 5:**
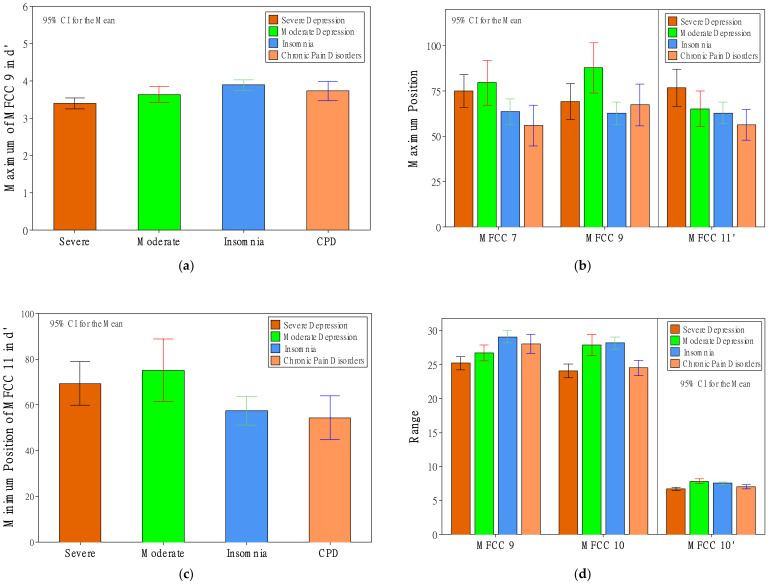

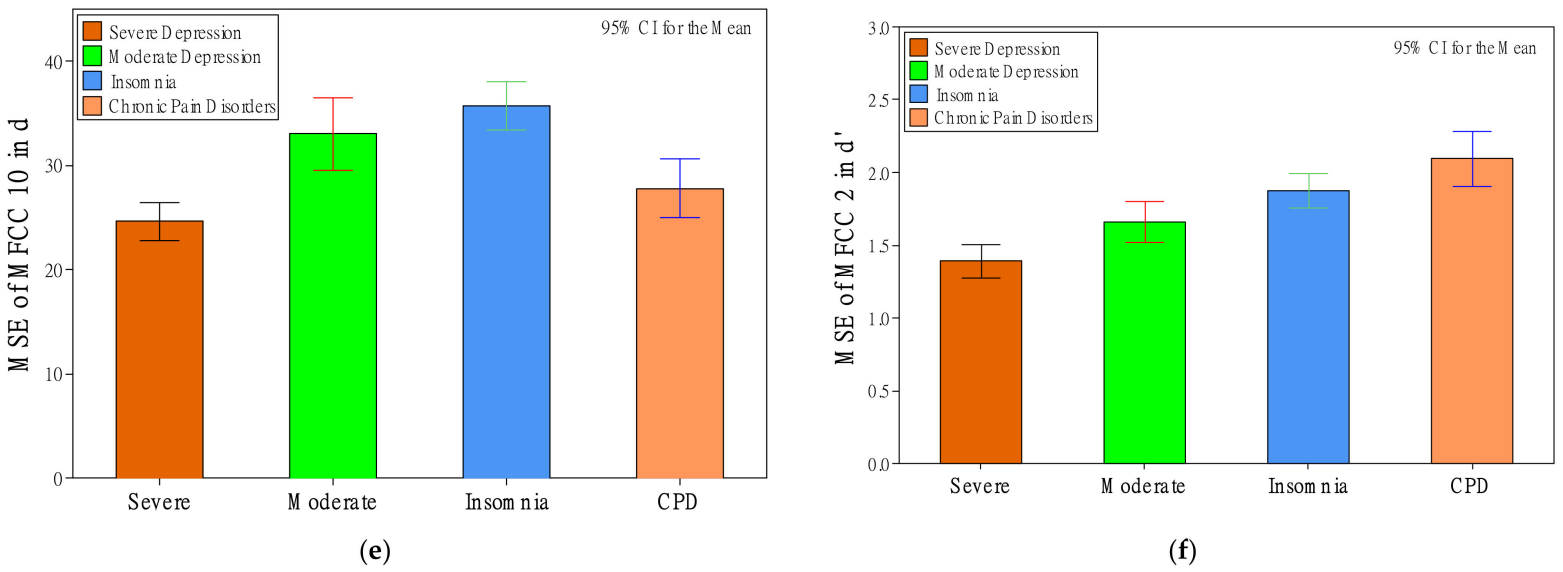
The effect of patient status on maximum value (**a**), maximum position (**b**), minimum position (**c**), range (**d**), and MSE (**e**,**f**).

Other voice features that showed relatively less significant relationships (between one and two for either *d* or *d’* features) were SD, skewness, maximum value, maximum position, minimum position, range, and MSE. Regarding the SD features, as shown in Figure 4e, the severe-depression group had the lowest SD value of MFCC 10 and had the lowest SD values of MFCCs 2′ and 10′ in *d’* features. The SD value of MFCC 2′ showed an ideal trend in that SD values were significantly different among the four groups. The values increased from severe to moderate, insomnia, and then CPD. Regarding the skewness features, as shown in Figure 4f, the severe-depression group had the greatest skewness value of MFCC 2′. Regarding the maximum-value features, as shown in Figure 5a, the severe-depression group had the lowest maximum value of MFCC 9′. Regarding the maximum-position features, as shown in Figure 5b, there was a trend in that depression patients had greater maximum-position values, especially MFCC 7, compared to groups of insomnia and CPD. The moderate-depression group had the greatest maximum-position value of MFCC 9. The severe-depression group had the greatest maximum-position value of MFCC 11′. Regarding the minimum-position features, as shown in Figure 5c, again, depression patients had greater minimum-position values of MFCC 11′ compared to groups of insomnia and CPD. Regarding the range features, as shown in Figure 5d, the severe-depression group had the lowest range values of MFCCs 9, 10, and 10′. Regarding the MSE features, as shown in Figure 5e, the severe-depression group had the MSE range values of MFCC 10′. However, the MSE value of MFCC 2′, as shown in Figure 5f, showed an ideal trend in that MSE values were significantly different among the four groups. The values increased from severe to moderate, insomnia, and then CPD.

### 3.3. Effects of Gender and Interaction Effects of Status and Gender

Regarding RMS, ZCR, F0, and HNR, gender had significant effects (*p*-value < 0.05) in four *d* features and five *d’* features, as shown in Table 2. Among *d* features, significant relationships mainly occurred in the ZCR category, comprising ZCR-SD, ZCR-slope, and ZCR-MSE features. The other significant relationship was the minimum value of HNR. Among *d’* features, ZCR-SD and ZCR-MSE features were significantly affected by gender as well. The other significant gender effects were on RMS-skewness, F0-mean, and HNR-mean features.

Regarding MFCC features, gender significantly affected 20 *d* features and 15 *d’* features. For both *d* features and *d’* features, most of the gender effects (32 out of 35) occurred in MFCCs 7–12. MFCC 1-range, MFCC 1-MSE in *d’* features, and MFCC 5′-mean in *d’* features were the only three exceptions.

As shown in Table 3, there was no significant interaction effect of status and gender on RMS-, ZCR-, F0-, or HNR-related features. There was only one interaction effect in *d* features and eight in *d’* features.

## 4. Discussion

### 4.1. Voice Features Show the Differences among Patient Groups

This study showed the differences in voice features among patients with severe depression, moderate depression, insomnia, and CPD. To facilitate the comparison of our results with previous findings, Table 4 summarizes the relationships between patient status and critical voice features and how these voice features help in discriminating among the four groups of patients.

Surprisingly, regarding RMS, ZCR, F0, and HNR features, there were no significant status effects on any mean- or range-related features. As shown in Table 4, previous studies reported that compared to other emotions (e.g., anger, happiness, and fear), sadness results in lesser values of RMS-mean [43,44], RMS-range [43], F0-mean [43], F0 range [43], and resonant voice quality (i.e., high HNR-mean) [44]. However, this study did not find significant effects regarding these phenomena. The main reason could be due to different comparisons. Except for Kiss and Vicsi [45], who compared depression with healthy patients, previous studies compared sadness with other distinguishable emotions, such as anger, happiness, and fear. However, the four groups of patients had relatively fewer emotional differences in this study. Nevertheless, this study found statistical properties other than mean and range in *d* features that can help discriminate depression patients from insomnia patients and patients with CPD. Our findings show that depression groups had greater RMS-skewness, RMS-kurtosis, F0-skewness, and F0-kurtosis values. RMS-skewness and RMS-kurtosis features discriminated between groups of severe depression and CPD and discriminated these two groups from the other two groups (see Figure 1 and Table 4). Moreover, F0-skewness and F0-kurtosis features discriminated between severe- and moderate-depression patients and discriminated these two groups from the other two groups (see Figure 2 and Table 4). Furthermore, this study showed that depression groups had lower slope values of F0 and HNR, helping discriminate severe-depression, moderate-depression, and insomnia groups (see Figure 3 and Table 4). Note that there were no significant differences between the severe-depression patients and CPD patients.

The status effects on MFCC features provide more help in discriminating patient groups. Taguchi, et al. [46] compared 16 original descriptors (mean values of RMS, ZCR, F0, HNR, and 12 MFCC) of 36 major depression patients and 36 healthy controls, and reported that MFCC 2-mean was the only feature significantly affected by depression. The MFCC 2-mean value was relatively higher in depression patients than in the control group. Although we did not find a significant effect on MFCC 2-mean, we found that MFCC 2′ provides the most helpful voice features among all the 384 voice features. As shown in Table 4, MFCC 2′-SD, MFCC 2′-offset, MFCC 2′-slope, and MFCC 2′-MSE were ideal voice features that can discriminate between the four patient groups. These features were significantly different among the four groups. The values increased (i.e., MFCC 2′-SD, MFCC 2′-offset, and MFCC 2′-MSE) or decreased (i.e., MFCC 2′-slope) from severe to moderate, insomnia, and then CPD.

Other than MFCC 2, we further found status effects on other MFCC features. As for the rest of the features, we will attempt to discuss them according to how they help in discriminating between our patients. First, as F0-slope and HNR-slope features, MFCC 9′-maximum, MFCC 10-SD, MFCC 10-MSE, MFCC 10′-SD, and MFCC 10′-range were helpful voice features for discriminating among severe-depression, moderate-depression, and insomnia groups. Again, these features may confuse CPD patients with one of the other three groups of patients. Second, MFCC 12-offset and MFCC 11′-maximum features discriminated between groups with severe depression and CPD and discriminated these two groups from the other two groups. Third, MFCC 9-range discriminated between groups of severe depression and moderate depression and discriminated these two groups from the other two groups. Fourth, MFCC 3′-offset, MFCC 3′-slope, MFCC 6′-offset, and MFCC 12′-offset discriminated depression patients from the other two groups and discriminated insomnia patients and patients with CPD from each other as well. Fifth, MFCC 5-kurtosis, MFCC 7-maximum value, and MFCC 11′-minimum position discriminated depression patients from the other two groups. However, this category of features could not discriminate between insomnia patients and patients with CPD. Last, there were many helpful voice features for discriminating patients with CPD from depression and insomnia. Original descriptors were all offset-related, including MFCC 1-offset, MFCC 4-offset, MFCC 7-offset, and MFCC 12-offset. De-differentiated descriptors were mean- and offset-related, including MFCC 3′-mean, MFCC 5′-mean, MFCC 7′-mean, MFCC 12′-mean, MFCC 3′-offset, and MFCC 12′-offset.

Solomon, Valstar, Morriss, and Crowe [19] compared differences in original MFCC descriptors between depression and healthy participants when they were asked about a deeply emotional topic to describe their experiences with depression. As shown in Table 4, they reported that the mean of MFCC 5, maximums of MFCCs 4, 5, 8, and 10, and minimums of MFCC 5 were important features between the two groups. Although the critical features reported by Solomon, Valstar, Morriss, and Crowe [19] are not in line with our findings, both studies showed that mean- and SD-related MFCC *d* features may not be effective voice features for determining depression. Of course, the different results might be due to different comparisons and experimental settings.

### 4.2. Considerations of Gender Effects

The analyses of gender effects provide further considerations while selecting voice features for discriminating between depression and insomnia. Regarding RMS, ZCR, F0, and HNR, gender had significant effects (*p*-value < 0.05) on four *d* features and five *d’* features (Table 2). While previous studies reported gender effects on the pitch [26,27], voice quality [27], and pitch range [28], we found gender effects on ZCR, HNR (i.e., voice quality), and certain MFCCs (related to specific ranges of pitches). The discrepancy might be due to insufficient and unbalanced numbers of both genders in status groups. As mentioned above, although *d’* features were proposed by Cao, Xu, and Liu [42] to reduce individual differences, they were not effective in eliminating the effects of gender. Instead, de-differentiated descriptors help differentiate between gender differences with RMS, ZCR, F0, and HNR features. After de-differentiated processing, RMS-skewness, ZCR-MSE, F0-mean, and HNR-mean were additional descriptors that were significantly affected by gender. Regarding MFCC features, as shown in Table 2 and Table 3, there were 20 and 15 significant main effects of gender on *d* and *d’* features, respectively, and 1 and 8 significant interaction effects of gender and status on *d* and *d’* features, respectively. Although the distribution of these significant effects was irregular, most of these main and interaction effects (38 out of 44) occurred in MFCCs 7–12 for both *d* features and *d’* features.

While the primary purpose of selecting critical voice features is to discriminate between patient groups, but not gender, the selection of a voice feature significantly affected by both status and gender may reduce discrimination effectiveness. Hence, two strategies are suggested for selecting critical voice features for developing discriminating models. The first strategy is to exclude the voice features significantly affected by gender to avoid interference. The second strategy is to develop individual discriminating models for female and male patients, respectively.

### 4.3. Contributions and Implications

This preliminary study attempted to discourse on potential voice features for discriminating among the patients with severe depression, moderate depression, insomnia, and CPD. With six severe-depression patients, four moderate-depression patients, ten insomnia patients, and four patients with CPD, the statistical analyses show many significant patient status effects on voice features that were computed by openSMILE, IS09_eotion [41]. We report speech differences between depression and insomnia that were previously scarce in the literature, and our results support the hypothesis that there are voice features that help distinguish between depression and insomnia.

While previous findings mainly focus on mean- and range-related features [43,44], or even ignore the relationships between human status and voice features while developing artificial intelligence (AI) models [47,48,49], this study assessed more comprehensive relationships between patient status and voice features. While the ultimate objective was to help clinical diagnoses, studies on the rationales behind significant relationships allow us to select critical features (as shown in Table 4) for further developing AI models confidently.

### 4.4. Limitations and Future Research

Although our analyses explained the effects of depression on certain voice features, the limited number of participants recruited in this study may not provide convincing evidence, even although we attempted to include four CPD patients in the analyses to represent a control group showing no depression and no insomnia. The number the patients and the narrow diversity of symptoms of this group make the generalization of the results difficult. However, despite these limitations, this preliminary study showed the potential of this approach and is encouraging for future studies recruiting an adequate number of patients for each group. With well-selected voice features and sufficient data, we expect the development of AI models for discriminating between patients for use in clinical recommendations. Although this study only discussed the statistically significant relationships with a *p*-value < 0.05, the effects with significance < 0.1 could be tested as well when developing models. Table 1, Table 2 and Table 3 show these voice features with a symbol of ‘^’.

## 5. Conclusions

This preliminary study aimed at disclosing critical voice features for discriminating between depression and insomnia using statistical analyses. With six severe-depression patients, four moderate-depression patients, ten insomnia patients, and four CPD patients, the statistical results show the patient status effects on certain voice features, demonstrating the potential to develop discriminating models of depression and insomnia using voice features. The findings encourage recruiting an adequate number of patients to confirm these voice features and increasing the number of the data in future studies. The ultimate goal was to apply critical voice features with adequate data to develop a quantitative method to help discriminate between depression patients and insomnia patients.

## Figures and Tables

**Table 1:** Effects of patient status on 384 voice features.

Effects		Original Descriptors (*d*)												De-Differentiated Descriptors (*d’*)											
		Mean	SD	Skewness	Kurtosis	Max Value	Min Value	Max Position	Min Position	Range	Offset	Slope	MSE	Mean	SD	Skewness	Kurtosis	Max Value	Min Value	Max Position	Min Position	Range	Offset	Slope	MSE
RMS				*	*																				
ZCR			**^**			**^**					**^**														**^**
F0		**^**		●	*							*					**^**								
HNR		**^**										*													
MFCC	1										*														
	2				**^**										*	*							●	*	*
	3													*			*						●	■	
	4										*														
	5				●									*			*								
	6															*				**^**	*		*	**^**	
	7							*			*			*											
	8																								
	9		**^**					*		*					**^**			*				**^**	**^**		**^**
	10		*		*		**^**			*	**^**		*		*		●	**^**	**^**			*	**^**		**^**
	11										**^**				**^**		*			*					**^**
	12				●						*			●									**^**	*	

^: *p* < 0.1; *: *p* < 0.05; ●: *p* < 0.01; ■: *p* < 0.001.

**Table 2:** Effects of patient gender on 384 voice features.

Effects		Original Descriptors (*d*)												De-Differentiated Descriptors (*d’*)											
		Mean	SD	Skewness	Kurtosis	Max Value	Min Value	Max Position	Min Position	Range	Offset	Slope	MSE	Mean	SD	Skewness	Kurtosis	Max Value	Min Value	Max Position	Min Position	Range	Offset	Slope	MSE
RMS																*	**^**								
ZCR			*									*	*		*			**^**				**^**			●
F0														*		**^**									
HNR							*		**^**					*		**^**							**^**		
MFCC	1		**^**			**^**				*			*												
	2				**^**																		**^**		
	3																								
	4							**^**																	
	5													*									**^**		
	6									**^**			**^**					**^**				**^**			
	7	*				●		*			**^**	**^**		*							**^**		*	*	
	8																								
	9	*			**^**	*		*			*					*	*	**^**					●	*	
	10	*	**^**				●	●			●			●			**^**		*				*	*	
	11	*			●	●						●		**^**										**^**	
	12		*	**^**						*			*			■	*		●			**^**			

^: *p* < 0.1; *: *p* < 0.05; ●: *p* < 0.01; ■: *p* < 0.001.

**Table 3:** Interaction effects of patient status and gender on 384 voice features.

Effects		Original Descriptors (*d*)												De-Differentiated Descriptors (*d’*)											
		Mean	SD	Skewness	Kurtosis	Max Value	Min Value	Max Position	Min Position	Range	Offset	Slope	MSE	Mean	SD	Skewness	Kurtosis	Max Value	Min Value	Max Position	Min Position	Range	Offset	Slope	MSE
RMS																									
ZCR																									
F0																									
HNR																									
MFCC	1																								
	2															*							**^**		
	3												**^**										*	*	
	4																								
	5																								
	6																								
	7													*											
	8							**^**			**^**														
	9							*															*		
	10						**^**				**^**					**^**							*	**^**	
	11																**^**								
	12													*									**^**	●	

^: *p* < 0.1; *: *p* < 0.05; ●: *p* < 0.01; ■: *p* < 0.001.

**Table 4:** Comparisons of this study and other studies on the depression effects on voice features.

Feature Category	Original Descriptor (*d*)				De-Differentiated Descriptor (*d’*)	
	Emotion Study	Depression Study	This Study		This Study	
RMS	Mean▼ [43,44] Range▼ [43]	–	Skewness▲	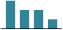	–	
			Kurtosis▲	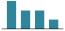	–	
ZCR	–	–	–		–	
F0	Mean▼ [43] Range▼ [43]	Mean▼ [45]	Skewness▲	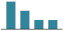	–	
			Kurtosis▲	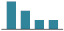	–	
			Slope▼	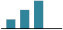	–	
HNR	Mean▲ [44]	–	Slope: ▼	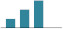	–	
MFCC 1	–	–	–		–	
MFCC 2	–	Mean▲ [46]	–		SD’▼	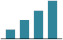
			–		Offset’▼	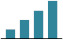
			–		Slope’▲	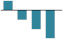
			–		MSE’▼	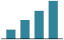
MFCC 3	–	–	–		Offset’	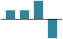
			–		Slope’	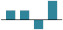
MFCC 4	–	Max? [19]	–		–	
MFCC 5	–	Mean? [19] Max? [19] Min? [19]	Kurtosis▲	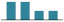	–	
MFCC 6	–	–	–		Offset’	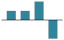
MFCC 7	–	–	Max P.▲	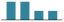	–	
MFCC 8	–	Max? [19]	–		–	
MFCC 9	–	–	Range▼	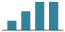	Max’▼	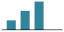
MFCC 10	–	Max? [19]	SD▼	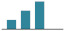	SD’▼	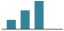
			MSE▼	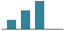	Range’▼	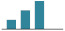
MFCC 11	–	–	–		Max’▲	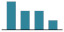
			–		Min P’▲	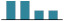
MFCC 12	–	–	Offset▲	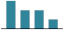	Slope’▲	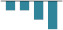

Note: ▼: depression had lesser values; ▲: depression had lesser values; ?: no comparison; bars from left to right in a bar chart show groups of severe-depression, moderate-depression, insomnia, and CPD patients, respectively, and different bar lengths show statistical differences among the four groups of patients.
